# Using a Portable Gait Rhythmogram to Examine the Effect of Music Therapy on Parkinson’s Disease-Related Gait Disturbance

**DOI:** 10.3390/s21248321

**Published:** 2021-12-13

**Authors:** Emiri Gondo, Saiko Mikawa, Akito Hayashi

**Affiliations:** 1 Department of Rehabilitation, Juntendo University Graduate School of Medicine, 2-1-1 Hongo, Toyko 113-8421, Japan; emirigondo@gmail.com; 2 Department of Rehabilitation, Juntendo University Urayasu Hospital, 2-1-1 Tomioka, Urayasu 279-0021, Japan; sa-aiba@juntendo.ac.jp

**Keywords:** portable gait rhythmogram, 3-D gait analysis, music therapy, Parkinson’s disease, gait disturbance

## Abstract

External cues improve walking by evoking internal rhythm formation related to gait in the brain in patients with Parkinson’s disease (PD). This study examined the usefulness of using a portable gait rhythmogram (PGR) in music therapy on PD-related gait disturbance. A total of 19 subjects with PD who exhibited gait disturbance were evaluated for gait speed and step length during a 10 m straight walking task. Moreover, acceleration, cadence, and trajectory of the center of the body were estimated using a PGR. Walking tasks were created while incorporating music intervention that gradually increased in tempo from 90 to 120 beats per minute (BPM). We then evaluated whether immediate improvement in gait could be recognized even without music after walking tasks by comparing pre- (pre-MT) and post-music therapy (post-MT) values. Post-MT gait showed significant improvement in acceleration, gait speed, cadence, and step length. During transitions throughout the walking tasks, acceleration, gait speed, cadence, and step length gradually increased in tasks with music. With regard to the trajectory of the center of the body, we recognized a reduction in post-MT medio-lateral amplitude. Music therapy immediately improved gait disturbance in patients with PD, and the effectiveness was objectively shown using PGR.

## 1. Introduction

Parkinson’s disease (PD) is a neurodegenerative condition among elderly populations [1] that develops through the degeneration of dopaminergic neurons in basal ganglia, causing a deficiency of such neurons [2]. The main symptoms of PD include resting tremors, rigidity, bradykinesia, postural instability [3], and gait disturbance—one of the most frequent and intractable motor disturbances [4]. Gait disturbance related to PD is an evolving condition with different patterns [5], such as reduced step length [6], slow speed, shuffling steps [7], and freezing of gait. Furthermore, gait disturbance not only decreases mobility and increases the risk of falling [8] but also restricts the functional independence and quality of life of people with PD (PwP) [9].

Although PD has been primarily treated through antiparkinsonian drugs and surgery with deep brain stimulation, evidence has shown that combining different rehabilitation approaches, such as physiotherapy, occupational therapy, and speech therapy, with antiparkinsonian drugs and surgical treatment can be more effective [10,11,12]. Apart from the mentioned therapies, music therapy, and a cue-based strategy that uses external sound rhythms to evoke walking rhythms in the brain [4,13], has also attracted attention in recent years. Among the various external cues (auditory, visual, and antennal cues), rhythmic auditory stimulation has been the most effective for gait disturbance treatment in PwP [3]. Moreover, reports have shown that gait training matched to metronomic rhythms can increase gait speed [14,15].

Over the years, there has been remarkable development in wearable devices and methods for investigating gait. The current study utilized a portable gait rhythmogram (PGR) that uses inertial sensors to evaluate three-dimensional (3-D) changes in the walking trajectory of the center of the body. The major advantages of inertial sensors include their small size, low cost, and long operating life, which allow for the unobtrusive monitoring of the walking pattern without interfering with the natural movement [16]. Moreover, a PGR allows us to easily monitor patient progress by drawing and visualizing the walking trajectory using gait data instead of simply comparing numerical values [16]. An objective and quantitative gait analysis system could, therefore, potentially improve the current practice (i.e., semi quantitative gait evaluation), which could aid in the diagnosis, symptom monitoring, therapeutic management, rehabilitation, fall risk assessment, and prevention of PD [17]. In addition to the medical field, a PGR can also be used in the field of music therapy, where it is difficult to quantify its overall effect on various diseases and there is still limited evidence-based research [18].

To investigate the effectiveness of music therapy as a rehabilitation approach for gait disturbance in PD, the current study utilized gait training with and without rhythmic auditory stimulation and examined whether immediate improvements occurred after training by evaluating the walking speed, acceleration, cadence, stride length, and walking trajectory of the center of the body.

Note that this is a small, open-label study with no long-term effects, therefore this is a pilot study describing the potential use of this method.

## 2. Materials and Methods

### 2.1. Subjects

A total of 19 PwP with mild gait disturbance (6 males and 13 females; mean age, 74.0 ± 6.7; H&Y, 2 or 3; duration, 6.0 ± 5.5 years; UPDRS-III, 17.3 ± 4.7) were included in this study. They had a gait score of 0 to 2 in the UPDRS-III and were able to walk without assistance. On examination, no one had freezing and no one had a high probability of falling. Regardless of the medication taken by the subjects, conventional treatments were provided without any changes.

### 2.2. Gait Analysis

**Figure 1:**
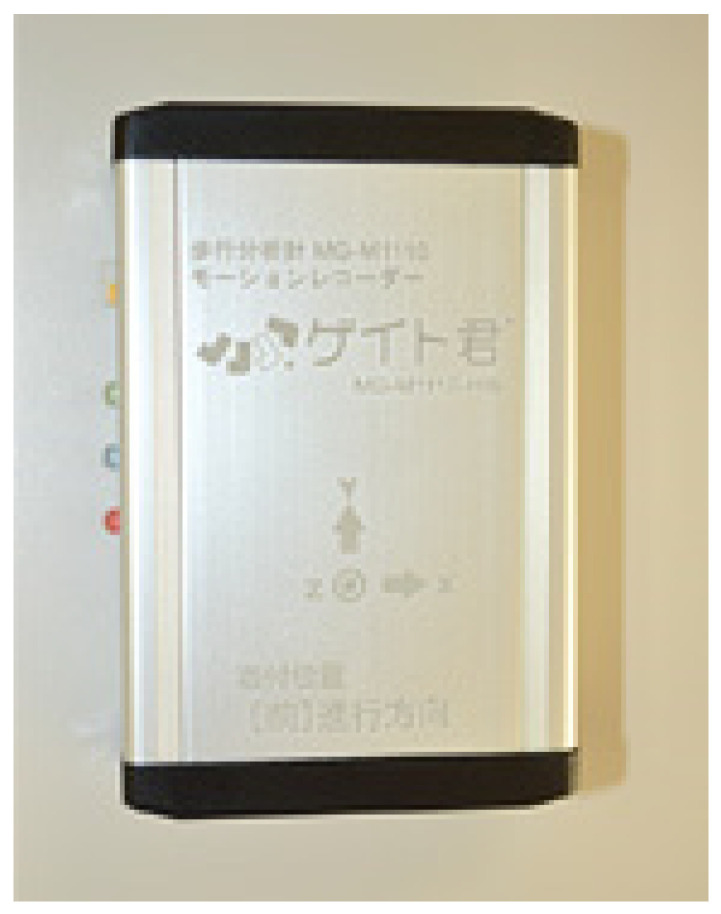
Portable gait rhythmogram (MG-M 1110, LSI Medience Corporation, Tokyo, Japan). Size = 8 × 6 × 2 cm. Weight = 80 g.

Gait was analyzed using a PGR (MG-M 1110, LSI Medience Corporation, Tokyo, Japan), a small device (8 × 6 × 2 cm, weight; 80 g) that houses an accelerometer (Figure 1). As reported previously [19], gait-induced acceleration is extracted from limb and trunk movements using an automatic gait detection algorithm (“pattern matching method”), allowing for the 3-D measurement (*a_x_*, *a_y_*, *a_z_*) of acceleration associated with voluntary limb and trunk movements, as well as acceleration induced by heel strike and toe-off when walking. As reported in detail previously [19], based on the “pattern matching method”, the acceleration vectors associated with stepping can be distinguished from those associated with other limb and trunk movements or with unexpected artifacts. First, attention is focused on a relatively strong signal region (e.g., *a* > 1 m/s^2^) in the acceleration time series, and a 3-D template wave (*a_x_*, *a_y_*, *a_z_*) with a duration of about 0.5 s is arbitrarily chosen. Then, the cross-correlation *CC*(*t*) between this wave and another wave with a time shift *t* chosen from the whole time series is computed using the following formula:

$$CC\left(t\right)=\frac{\frac{1}{p}\sum_{i=1}^{p}\left[a_{x}\left(i\right)a_{x}\left(i+t\right)+a_{y}\left(i\right)a_{y}\left(i+t\right)+a_{z}\left(i+t\right)\right]}{{\left\{\frac{1}{p}\sum_{i=1}^{p}\left[a_{x}{\left(i\right)}^{2}+a_{y}{\left(i\right)}^{2}+a_{z}{\left(i\right)}^{2}\right]\right\}}^{\frac{1}{2}}{\left\{\frac{1}{p\sum_{i=1}^{p}\left[a_{x}{\left(i+t\right)}^{2}+a_{y}{\left(i+t\right)}^{2}+a_{z}{\left(i+t\right)}^{2}\right]}\right\}}^{\frac{1}{2}}}$$

where *t* is the time index and *p* is the length of the template wave. If the acceleration change is caused by gait motion, the *CC*(*t*) peaks exhibit alternate changes in magnitude with time due to left/right body sway during walking. Additionally, the cycle and amplitude are measured from the gait-induced acceleration signals. Since gait accelerations correlate with floor reaction forces, the amplitude of gait accelerations is selected as an index of floor reaction forces [20].

**Figure 2:**
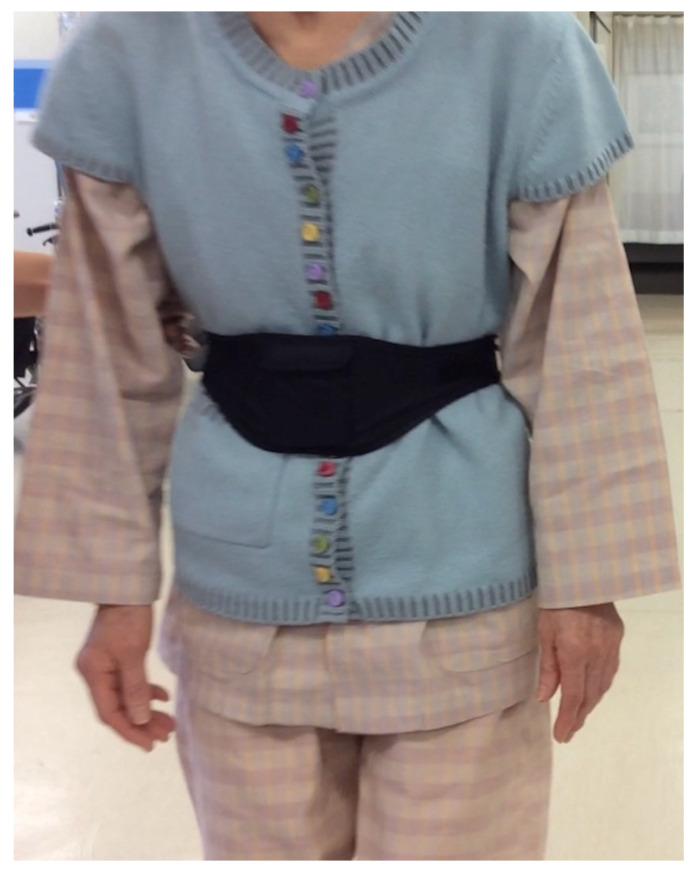
The device was secured at the center of subjects’ waists using a Velcro band.

The device was secured at the center of subjects’ waists using a Velcro band (Figure 2) and recorded the above signals at a sampling rate of 10 ms (100 Hz). The data were automatically stored on a microSD card. After transfer of the recorded data to a personal computer, the absolute values of acceleration vectors ($a;\;a^{2}=a_{x}{}^{2}+a_{y}{}^{2}+a_{z}{}^{2}$) were calculated offline and graphically displayed on the monitor [16,21]. With the subject standing in the anatomical position, the three acceleration axes (X, Y, and Z) were oriented in the mediolateral (ML), vertical (VT), and anteroposterior (AP) directions, respectively. Accordingly, positive X, Y, and Z values indicated leftward, upward, and forward acceleration, respectively.

Gait analysis was conducted in a large indoor space. All subjects were requested to walk back and forth in a 5 m straight line [22] without assistance, in accordance with seven common walking tasks (details provided later). An extra 1 m distance was added before and after the walkway to minimize the influence of acceleration and deceleration. The time a subject took to walk along the 5 m and 10 m walkways was determined from the event marker recordings. The timing and number of stride events during this time interval were identified from the 3D acceleration signal using an automated peak detection algorithm [21]. In addition, the 5 m walking time and step count were measured for each task by an experimenter. While the subject was walking, an experimenter with a stopwatch followed slightly behind [16]. Videos of walking were also taken to observe the walking pattern, posture, and swinging of the arms. Based on these data, the basic gait characteristics (gait speed, cadence, step length) were calculated for each subject. Next, the 3-D acceleration signal was filtered by a high-pass filter *sT*/(1 + *sT*) to remove slowly varying trends. The time constant was set at *T* = 0.7 s. Then, the acceleration magnitude *a_r_*(*t*) was calculated from the filtered components (*a_x_*(*t*) *a_y_*(*t*) *a_z_*(*t*)) by *a_r_*(*t*) = (*a_x_*(*t*)^2^ + *a_y_*(*t*)^2^ + *a_z_*(*t*)^2^)_0.5_ [22].

The common tasks were as follows: fast walking (walking as fast as possible without falling) (Task 1), self-paced walking with hand clapping (Task 2), walking in step with music at 90 beats per minute (BPM) (Task 3), 100 BPM (Task 4), 110 BPM (Task 5), and 120 BPM (Task 6), and fast walking again without music (Task 7). The instructions for each task were given as follows: “Please walk as fast as you can without falling”. for Task 1, “Please walk at your own pace. I clap at your pace”. for Task 2, “Next, music will be played. Please walk to the music”. for Task 3, “Another track will be played. Please walk to the music again.” for Tasks 4, 5, and 6, and “Next, there will be no music. Once again, please walk as fast as you can without falling”. for Task 7. Thereafter, pre-MT (Task 1) values for acceleration (gait force), gait speed, cadence, step length, and gait trajectory of the center of the body were compared to post-MT (Task 7) values to evaluate whether improvement in gait occurred immediately after walking tasks with music (from Task 3 to Task 6), even without music. The hand clapping in Task 2 was performed in rhythm with the subjects’ own pace and was observed by a separate experimenter. The music genres used in Tasks 3–6 included familiar classical music and Japanese traditional songs that matched metronomic rhythms (Japanese traditional song for BPM 90, classical music for BPM 100, Japanese traditional song for BPM 110, and classical music for BPM 120). The songs had no lyrics, and the melody was instrumentally edited by MIDI. The same tunes were used for all subjects. The music was played through the built-in speaker of a personal computer. Before starting the tasks, we played the music in fragments and checked if the subjects could hear it. All music was played at the same volume during the tasks. In each task, the four-beat metronome was played first and the participants were allowed to start walking at their own leisure after the music started.

### 2.3. Statistical Analysis

Significant differences between mean pre-MT and post-MT values of acceleration, gait speed, cadence, step length, and amplitude of the trajectory were evaluated using the paired *t*-test for normally distributed data. Statistical analysis was performed using StatPlus version 6.2.21 software for Macintosh (Apple Inc., Tokyo, Japan), with a *p* value of <0.05 indicating statistical significance.

Pre-MT and post-MT descriptive data were compared and analyzed using paired *t*-tests. An analysis of variance test was used to perform multiple comparisons among all seven tasks.

## 3. Results

**Figure 3:**
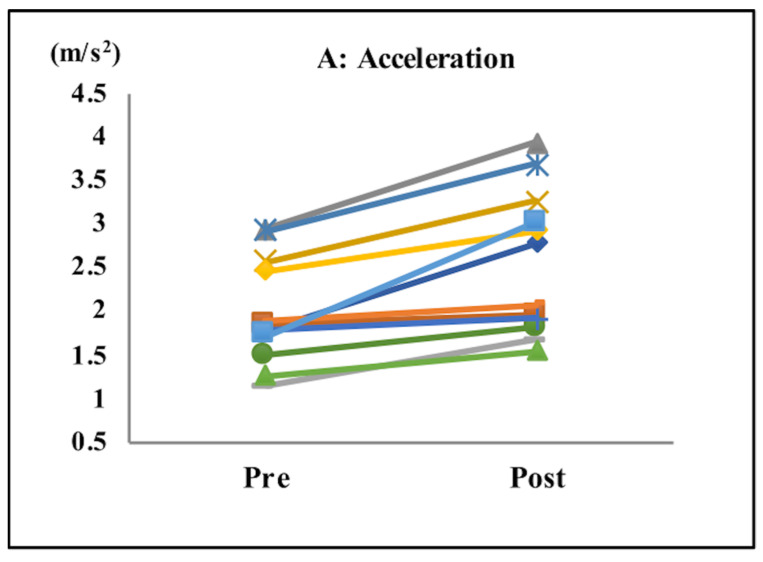

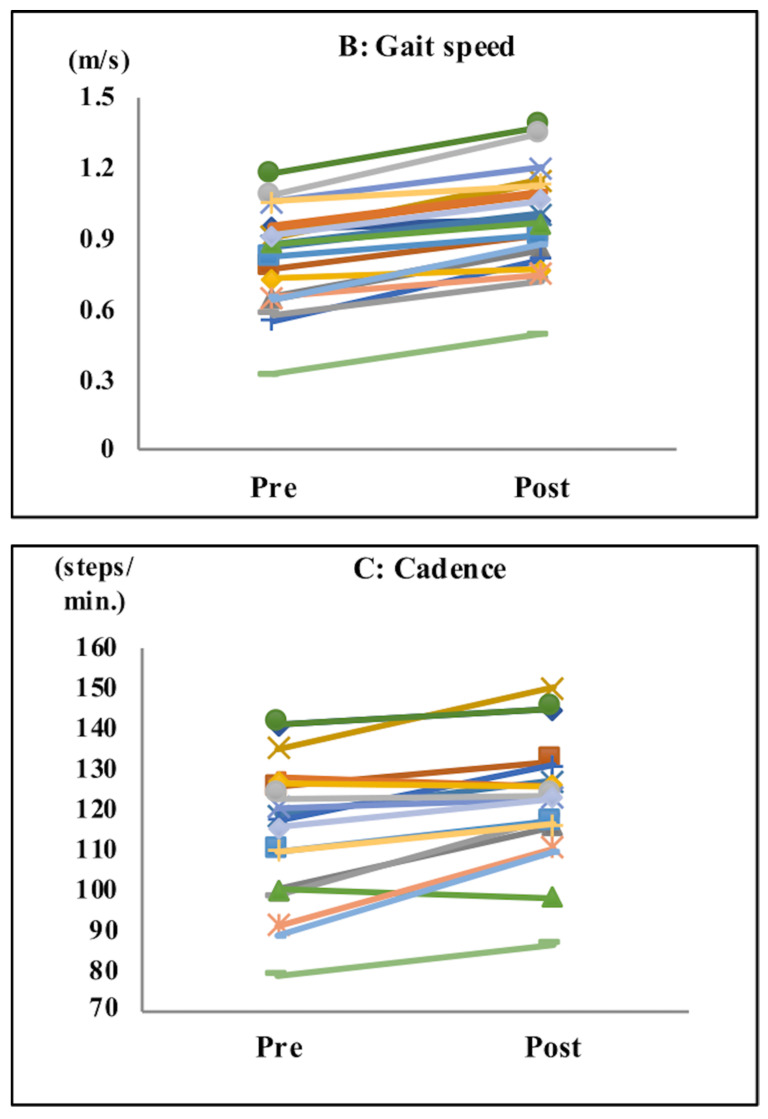

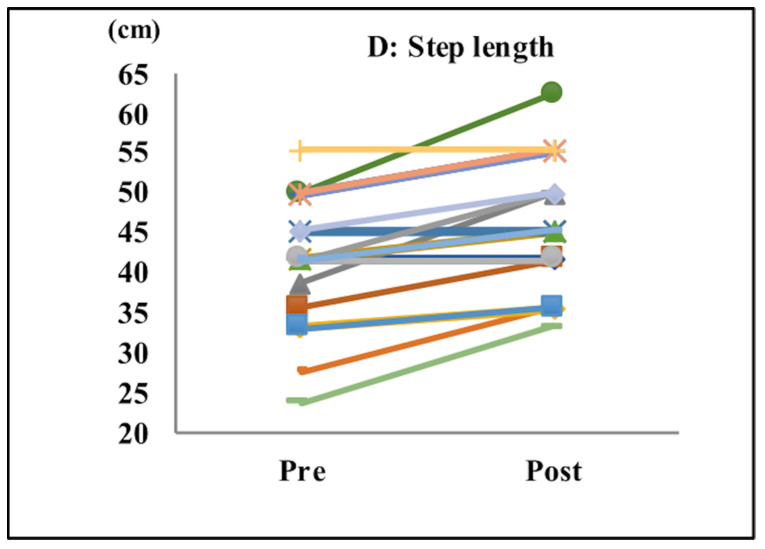
Gait parameters. Acceleration (**A**), gait speed (**B**), cadence (**C**), and step length (**D**). After the tasks with music, subjects showed significant improvements in their acceleration, gait speed, cadence, and step length.

After completing the tasks with music, subjects exhibited significant improvements in their acceleration (pre: 1.94 m/s^2^; post: 2.44 m/s^2^; *p* < 0.001; Table 1 and Figure 3A), gait speed (pre: 0.88 m/s; post: 0.97 m/s; *p* < 0.04; Table 1 and Figure 3B), cadence (pre: 117.3 steps/min; post: 122.9 steps/min; *p* < 0.0001; Table 1 and Figure 3C), and step length (pre: 41.55 cm; post: 46.70 cm; *p* < 0.0001; Table 1 and Figure 3D).

Additionally, we compared the pre/post changes in acceleration, gait speed, cadence, and step length between the two groups of H&Y 2 (4 subjects) and 3 (15 subjects), but there was no significant difference between the different H&Y levels. Furthermore, the same comparison was made between the two groups with a gait score of one point (12 subjects) and two points (6 subjects) on the UPDRS-III, but there was no significant difference between these two groups (the remaining subject had zero point for Gait on the UPDRS-III).

**Figure 4:**
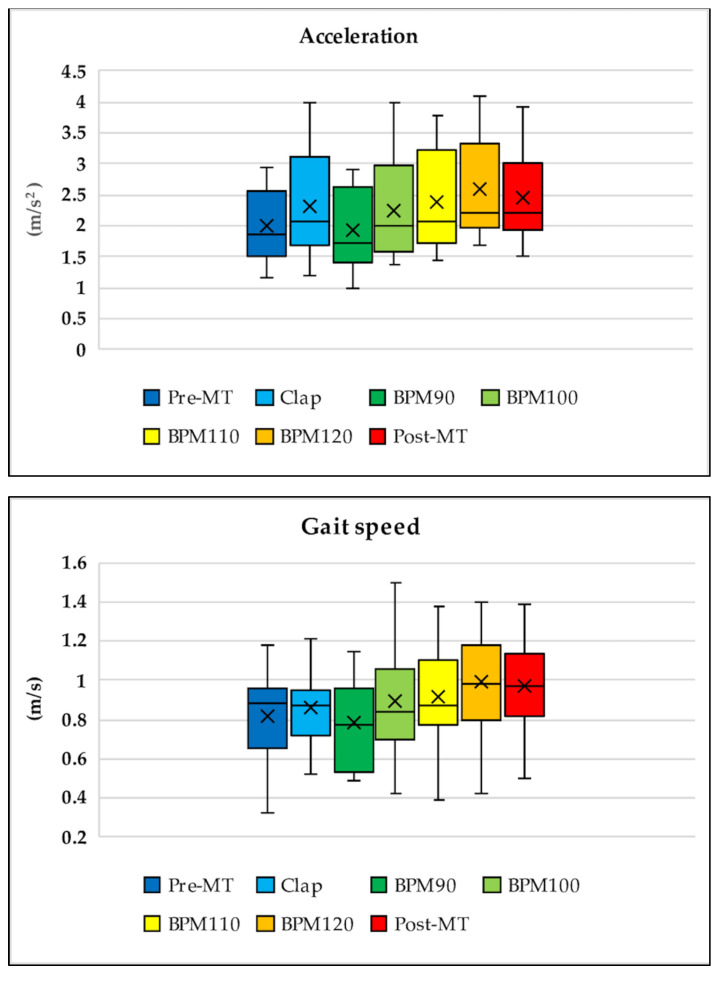

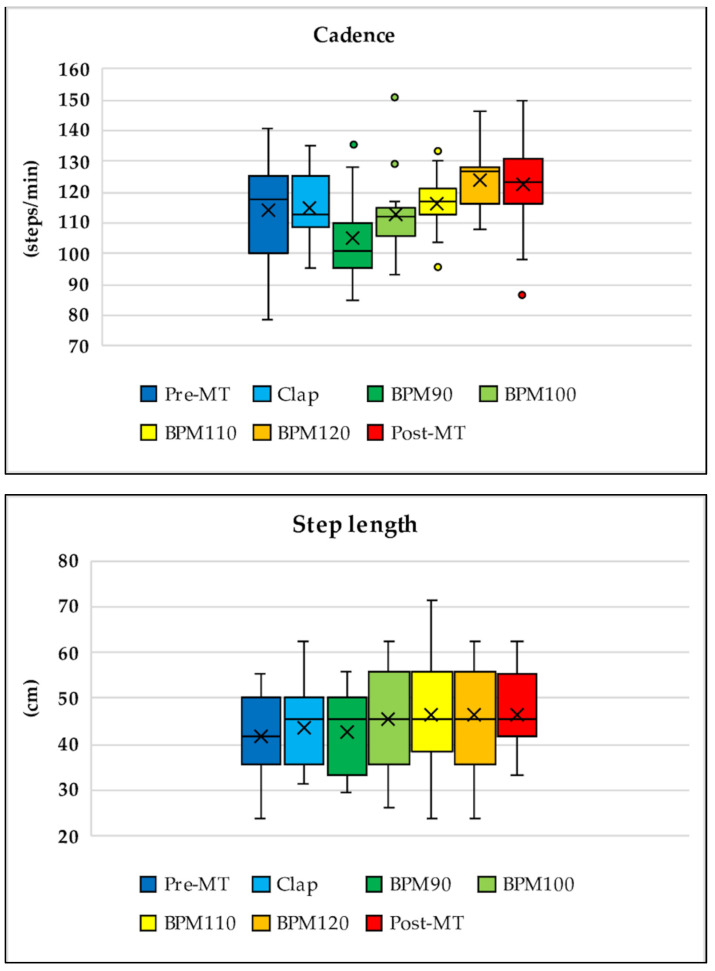
Transition throughout the entire walking task for acceleration, gait speed, cadence, and step length. During transitions in the walking tasks with music (from Tasks 3 to 6), values of acceleration, gait speed, cadence, and step length increased gradually. The best improvement was observed in Task 6 (music at 120 beats per minute (BPM)) and remained in Task 7 even without music. Walking speed in Task 3 (music at 90 BPM) was slightly slower than that in Task 2 (hand clapping while walking at the subjects’ own pace).

During transitions in all walking tasks with music (from Tasks 3 to 6), the values of acceleration (*p* < 0.05), gait speed (*p* < 0.08), cadence (*p* < 0.001), and step length (*p* < 0.6) gradually increased. The best improvements were noted during Task 6 (music at 120 BPM), with the effects remaining in Task 7 even without music (Figure 4). Walking speed in Task 3 (music at 90 BPM) was slightly slower than that in Task 2 (with hand clapping), which was performed at the subjects’ own pace. The rate of change in gait speed was −8.66% ± 3.31% for Task 3 (BPM 90), 3.82% ± 4.19% for Task 4 (BPM 100), 7.13% ± 4.57% for Task 5 (BPM 110), 16.04% ± 4.29% for Task 6 (BPM 120), and 15.48% ± 4.8% for Task 7 (post) (*p* < 0.001). The rate of change in acceleration was −16.83% ± 2.49% for Task 3 (BPM 90), −0.8% ± 4.46% for Task 4 (BPM 100), 4.04% ± 3.27% for Task 5 (BPM 110), 15.52% ± 5.56% for Task 6 (BPM 120), and 9.19% ± 4.44% for Task 7 (post) (*p* < 0.0001).

**Figure 5:**
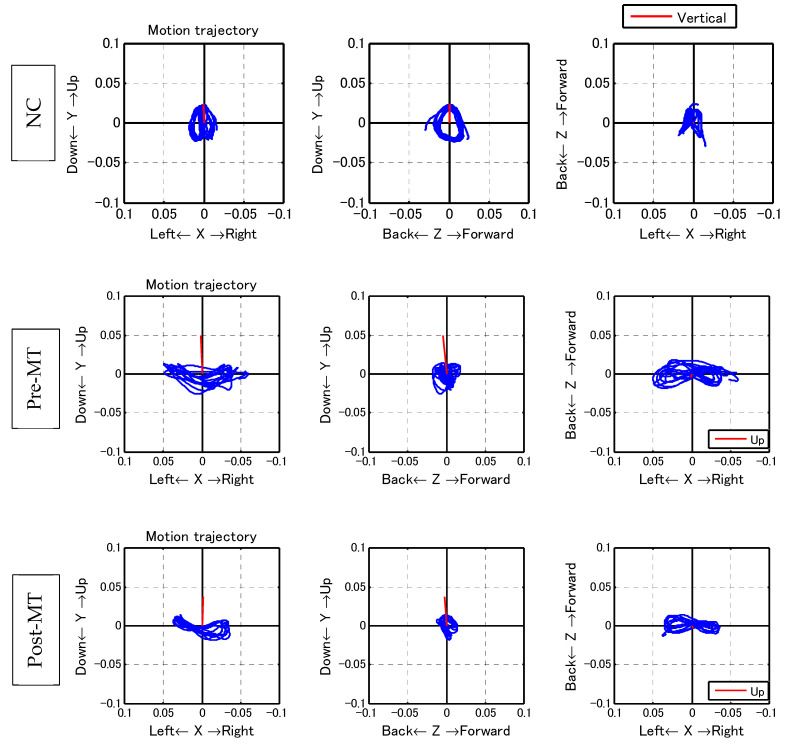
Walking trajectory. With regard to the trajectory of the center of the body, mediolateral (ML) amplitude, which is typically large for individuals with Parkinson’s diseases, decreased significantly. Left row: coronal plane; middle row: sagittal plane; right row: horizontal plane. Upper row: the walking trajectory of a healthy 70-year-old woman. The trajectory was symmetrical and forms a butterfly pattern in the coronal and horizontal plane. On the sagittal plane, the trajectory forms a symmetrical circle. Middle row: Pre-MT walking trajectory of one subject. Lower row: Post-MT walking trajectory of the subject shown in the middle row.

With regard to the trajectory of the center of the body, the ML amplitude, which is typically large for PwP [23], was reduced significantly (pre: 1.88 ± 0.98 cm; post: 1.71 ± 0.84 cm; *p* < 0.05; Table 2, Figure 5).

## 4. Discussion

On comparing walking before and after rhythmic auditory stimulation with music, the current study found that music therapy had immediate effects for gait disturbance in PwP. Accordingly, significant improvements were observed in acceleration, gait speed, cadence, and step length, suggesting the efficacy of music therapy in reducing gait disturbance associated with PD. As such, music therapy can be expected to be utilized in the rehabilitation of PwP, particularly those with gait disturbance.

Comparing the gait of PwP with that of normal controls in another study (NC; normal gait at 70–79 years) [22] showed that the former had clearly lower values for acceleration, gait speed, cadence, and step length compared to the latter. Accordingly, PwP and NC had pre-MT values of 1.94 ± 0.56 and 3.38 ± 0.16 m/s^2^ for acceleration, 0.88 ± 0.22 and 1.34 ± 0.01 m/s for gait speed, 109.99 ± 17.77 and 119.27 ± 2.05 steps/min for cadence, and 41.55 ± 8.38 and 67.43 ± 0.53 cm for step length, respectively. Music therapy was able to significantly improve gait disturbance in PD, which has been characterized as slow and small steps [5], a tendency observed herein.

**Figure 6:**
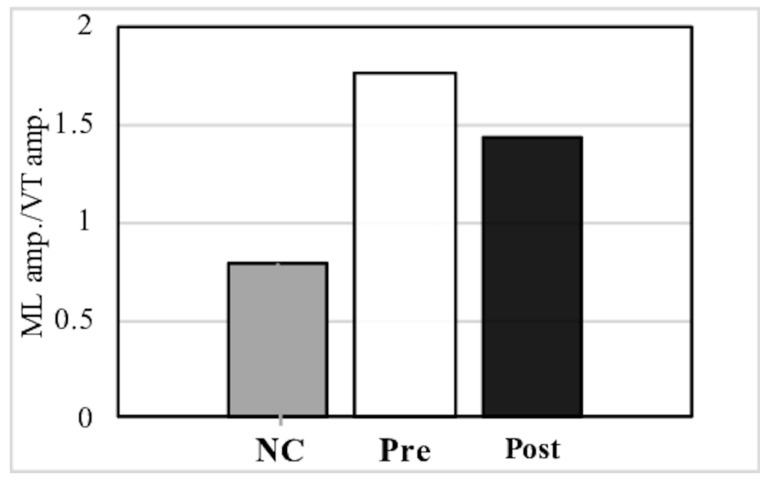

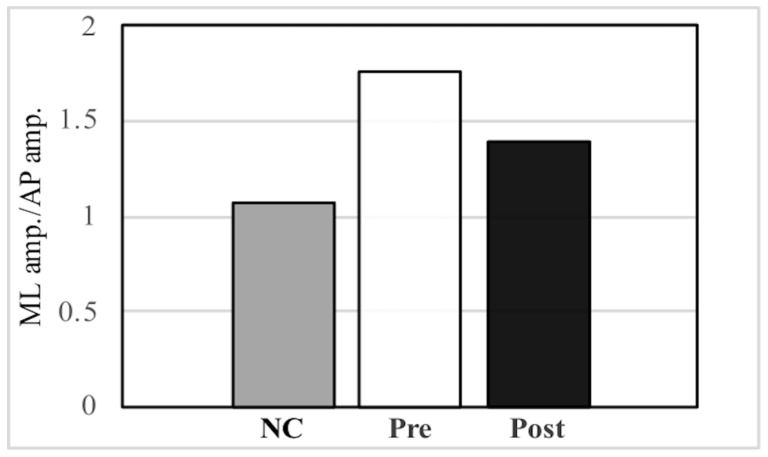
Comparison of the mediolateral (ML) amplitude/vertical (VT) amplitude ratio and ML amplitude/anteroposterior (AP) amplitude ratio between normal controls (NC) and pre- and post- music therapy individuals with Parkinson’s disease.

PwP also exhibit a gait that has large amplitude in the ML direction, which can be observed from the trajectory of the PGR (Table 2, Figure 5). After music therapy, however, the gait force increased, whereas the ML/VT amplitude and ML/AP amplitude ratios decreased (Figure 6). The use of a PGR allows us to observe the data objectively in a manner that both the patient and therapist can easily understand, which can be useful from the perspective of personal communication. Apart from gait analysis in PD, studies have utilized the trajectory of the PGR to evaluate total hip arthroplasty [16,21] and cerebral infraction [24]. PGR analysis clearly showed that the ML amplitude of PwP was larger than the VT and AP amplitude ratio among NC. Nonetheless, music therapy was able to improve gait trajectory of PwP as well.

Compared to NC, individuals with Parkinson’s disease had a larger ML amplitude ratio among the three axes, although this decreased post-MT.

The current study considers the following two factors to have caused the immediate effect. First, music can act as external stimuli that normalized the destabilized processes of internal rhythmic formation [14]. External rhythmic cues can serve as surrogate cues for impaired internal timing [12]. Second, music can promote a pleasurable feeling by activating the limbic system, which facilitates dopamine release [25,26]. Salimpoor et al. reported that the intense pleasure experienced when listening to music was associated with dopamine activity in the mesolimbic reward system, which includes both the dorsal and ventral striatum [27]. Thus, it can be hypothesized that the mechanism by which music therapy promoted its effects included both aforementioned factors, indicating its utility for rehabilitation. Although the immediate effects were examined in the current study, in the future, based on this method, the long-term effects could also be evaluated using PGR. Furthermore, since a fully charged PGR can achieve 70 h of continuous recording [20], it is possible to observe not only gait in the short term but also the diurnal variation of Parkinson’s disease patients and to detect freezing of gait.

Moreover, PD is commonly complicated by the existence of comorbid depression. In fact, Ito et al. [4] reported that simply listening to audio tapes at home without gait training for at least an hour daily over three to four weeks significantly decreased mean depression scale scores, suggesting that music could also be useful for improving depression as well as gait disturbance.

Rehabilitation plays a crucial role in maintaining quality of life associated with PD [4], the effects of which depend on the patients’ own motivation. Therefore, rehabilitation techniques that patients can voluntarily perform by themselves are needed. Hence, music therapy together with other rehabilitation approaches can be beneficial.

The effectiveness of various nonpharmacological interventions, such as Tai Chi, robot-assisted gait training, Lee Silverman Voice Treatment, dance, video games, and virtual reality exercises, have also been reported [11,28]. In particular, music and dance have potential advantages in terms of noninvasive treatment and easy applicability [29].

Nowadays, telemedicine for PD has been receiving increasing attention [30]. In fact, reports have shown that rehabilitation can be provided through telemedicine [31] and that music therapy could also be used in that field.

## 5. Conclusions

The current study indicated in detail that gait training incorporating music via rhythmic auditory stimulation can be effective for treating gait disturbance associated with PD. Moreover, the objective and visualized data obtained using a PGR can aid in treating PD-associated gait disturbance. The rehabilitation process through music is simple, noninvasive, nonpharmacological, and inexpensive. Therefore, we believe that music therapy will become more utilized for the daily rehabilitation of PD-associated gait disturbance.

## Figures and Tables

**Table 1:** Comparison of gait characteristics before and after music therapy (MT).

	Pre-MT	Post-MT
Acceleration (m/s^2^)	1.94 ± 0.56	2.44 ± 0.73 **
Speed (m/s)	0.88 ± 0.22	0.97 ± 0.22 **
Cadence (steps/min)	117.3 ± 17.74	122.9 ± 15.56 *
Step length (cm)	41.55 ± 8.38	46.70 ± 8.39 **

** *p* < 0.001, * *p* < 0.05.

**Table 2:** Changes in acceleration and amplitude over the three axes (mediolateral (ML), vertical (VT), and anteroposterior (AP)) before and after music therapy (MT).

	Acceleration (m/s^2^)		Amplitude (cm)	
	Pre-MT	Post-MT	Pre-MT	Post-MT
Total	1.94 ± 0.56	2.44 ± 0.73	-	
ML	0.96 ± 0.22	1.13 ± 0.27	1.88 ± 0.98	1.71 ± 0.84
VT	1.96 ± 0.39	1.57 ± 0.47	1.07 ± 0.30	1.19 ± 0.36
AP	1.10 ± 0.31	1.15 ± 0.41	1.07 ± 0.99	1.23 ± 0.80

## Data Availability

The data presented in this study are available on request from the corresponding author. The data are not publicly available due to subjects’ privacy.
